# Bioactive Potential of Elderberry (*Sambucus nigra* L.): Antioxidant, Antimicrobial Activity, Bioaccessibility and Prebiotic Potential

**DOI:** 10.3390/molecules28073099

**Published:** 2023-03-30

**Authors:** Ioana Mariana Haș, Bernadette-Emőke Teleky, Katalin Szabo, Elemer Simon, Floricuta Ranga, Zorița Maria Diaconeasa, Anamaria Lavinia Purza, Dan-Cristian Vodnar, Delia Mirela Tit, Maria Nițescu

**Affiliations:** 1 Doctoral School of Biomedical Sciences, University of Oradea, 410087 Oradea, Romania; 2 Institute of Life Sciences, University of Agricultural Sciences and Veterinary Medicine, 400372 Cluj-Napoca, Romania; 3 Department of Food Science and Technology, University of Agricultural Sciences and Veterinary Medicine, 400372 Cluj-Napoca, Romania; 4 Department of Pharmacy, Faculty of Medicine and Pharmacy, University of Oradea, 410028 Oradea, Romania; 5 Department of Preclinical–Complementary Sciences, University of Medicine and Pharmacy “Carol Davila”, 050474 Bucharest, Romania

**Keywords:** elderberry, polyphenols, bioaccessibility, gastrointestinal digestion, prebiotic potential

## Abstract

Due to its abundance of physiologically active ingredients, one of the oldest medicinal herbs, elderberry (EB) *Sambucus nigra* L., is beneficial for both therapeutic and dietary purposes. This study determined the bioaccessibility of the phenolic compounds and the prebiotic potential of the polyphenols from freeze-dried EB powder (FDEBP), along with the antioxidant and antimicrobial activities of this extract. The most significant phenolic compounds in black EB are represented by anthocyanins (41.8%), predominating cyanidin-sambubiosides and cyanidin-glucosides (90.1% of the identified anthocyanins). The FRAP assay obtained the highest antioxidant activity value (185 ± 0.18 μmol Fe^2+^/g DW). The most sensitive to the antimicrobial activity of the extract was proven to be *Staphylococcus aureus*, and *Pseudomonas aeruginosa* had the lowest minimum inhibitory concentration of 1.95 mg/mL. To determine the prebiotic potential of the polyphenols, the cell growth of five probiotic strains (*Lactobacillus plantarum*, *L. casei*, *L. rhamnosus*, *L. fermentum* and *Saccharomyces boulardii*) was tested. The influence on cell growth was positive for all five probiotic strains used. Overall, the most significant increase (*p* < 0.05) was recorded at 1.5% FDEBP, on *L. casei* with a growth index (GI) of 152.44%, very closely followed by GI at 0.5% and 1% concentrations. The stability of the total phenolic compounds through simulated gastronitestinal digestion was increased (93%), and the bioaccessibility was also elevated (75%).

## 1. Introduction

Since prehistoric times, plants have been used for food and as remedies [1]. In recent years, the interest in food supplements and functional foods, which contain bioactive plant-based compounds, has continuously increased. Numerous scientific studies were carried out using different methods and revealed the benefits of products rich in bioactive compounds [2,3] for preventing and treating certain diseases, including cardiovascular and metabolic diseases, pathologies responsible for millions of deaths worldwide [4,5,6,7].

An important source of bioactive compounds, relatively little studied compared to other berries, is black elderberry (EB), *Sambucus nigra* L.—also known as elder, black elder, European elder, European black elder, and common elder; it is part of the family Adoxaceae, genus Sambucus, being a common species. It has three subspecies: *S. nigra* ssp. *canadensis, S. nigra* ssp. *Cerulea* and *S. nigra* ssp. *nigra* L., the latter being the European elder [8,9,10]. Since ancient times, black EB has been used as a natural remedy in the treatment of various ailments: EB was used in the treatment of colds and flu, constipation or for its diuretic and anti-inflammatory effects [10]; elderflowers were used as a remedy for various respiratory and skin conditions, joint pain or for the diuretic effect [11]; even the elder leaves were used to treat various skin diseases [12].

The existence of phenolic constituents, which have a significant antioxidant effect and can therefore remove free radicals and combat oxidative stress, a factor contributing to the deterioration of the human body and the emergence of several diseases, is mainly responsible for the therapeutic properties of EB [13,14]. Through the wide variety of polyphenols contained, anthocyanins, flavonols, phenolic acids, and proanthocyanidins, EBs have proven their multiple beneficial effects, showing cardiovascular protection [15], antidiabetic properties [16], the ability to counteract obesity and metabolic dysfunction [17], antiviral and antibacterial activity [18], antioxidant capacity [19], antitumor potential [20], antidepressant action [21], and more recently, the prebiotic effect [22].

Phenolic compounds can deteriorate as a consequence of being exposed to light, oxygen, enzymatic activity, unfavorable pH condition, temperature, water, and metal ions; thus, their positive qualities may be modified [23]. For better comprehension and assessment of the potential biological characteristics of phenolic compounds, it is essential to confirm their stability and absorption in the digestive tract. Phenolic components are subjected to physiochemical alteration (due to pH, temperature, and digestive enzymes) in the gastrointestinal environment [22]. Yet, the bioaccessibility of dietary phenolic compounds during gastrointestinal digestion (GID) determines their therapeutic effects [24].

From this perspective, in order to evaluate the biological properties, along with the aspects regarding the chemical composition, this study determined the bioaccessibility of the phenolic compounds after GID and the prebiotic potential of the polyphenols from black EB from the spontaneous flora of Romania. Additionally, the antioxidant and antimicrobial action was tested. As far as we know, this is the first study of this type on black EB. The results provide new insights into advancing knowledge and research opportunities for the development of new nutraceutical or adjunctive strategies that use this product’s bioactive potential.

## 2. Results

### 2.1. Antioxidant Activity Analysis

The antioxidant activity of the EB extract (*S. nigra* L.) was analyzed with the help of four different assay methods (DPPH, ABTS, FRAP, and CUPRAC), and the results are presented in Table 1. The lowest value was obtained by ABTS, and the highest with the FRAP assay.

### 2.2. Antimicrobial Activity Assay

The antimicrobial activity of the lyophilized EB powder extract was evaluated on a total of seven strains containing Gram-positive and Gram-negative bacteria and yeasts. The lyophilized black EB powder extract shows antimicrobial activity on all tested microorganisms. The most sensitive to the activity of the extract was proven to be *S. aureus*, *P. aeruginosa* and the two yeasts, the lowest minimum inhibitory concentration (MIC) being 1.95 mg/mL. However, in the case of *S. enterica* and both strains of *E. coli,* the MIC was weaker in comparison with the other tested strains, as it presented an MIC of 3.91 mg/mL. The results are presented in Table 2.

### 2.3. Qualitative and Quantitative Analysis of the Extracts by HPLC-DAD-ESI-MS, before and after GID

The high-performance liquid chromatography (HPLC-DAD-ESI-MS) analysis of the extract from the powder obtained from lyophilized EB revealed the presence of 12 polyphenolic compounds belonging to the subclasses: anthocyanins, flavonols, hydroxycinnamic acids, and hydroxybenzoic acid derivatives.

Quantitative data show that the most significant amount of phenolic compounds in black EB is represented by anthocyanins, precisely 41.8%, predominating cyanidin-sambubiosides and cyanidin-glucosides, the two compounds constituting 90.1% of the identified anthocyanins. As for flavonols, they represented 25.5% of the total phenolic compounds in lyophilized EB. The HPLC-DAD analysis revealed the presence of quercetin derivatives (94%) and kaempferol. Rutin is the most present compound of this class, representing 75.7% of the total flavonols and 19.27% of the total phenolic compounds identified. Hydroxycinnamic acids were present in a proportion of 18.6%, and hydroxybenzoic acid derivatives in a proportion of 14.1%.

The total content of polyphenols in the analyzed extract was 41.28 mg/g of lyophilized EB powder, of which consisted 17.25 mg of anthocyanins, 10.51 mg of flavonols, 7.69 mg of hydroxycinnamic acid derivatives and 5.83 mg of hydroxybenzoic acid derivatives (Table 3).

### 2.4. The Bioaccessibility of Phenolic Compounds of Sambucus nigra L. Fruits during Simulated Digestion

The calculation of bioaccessibility was carried out according to the formula presented by Stefănescu et al. [25], which is:

BI (%) = (Phenolic content after gastrointestinal digestion (in vitro)/Phenolic content before digestion) × 100(1)

Then, the individual phenolic compound content was assessed HPLC-DAD-ESI-MS in the gastric and intestinal phases. The results are presented in Table 3, and as can be seen, the anthocyanin content, such as cyanidin-diglucoside and cyanidin glucoside, decreased from 1.20 ± 0.07 and 15.56 ± 0.19 to 1.03 ± 0.09 and 8.14 ± 0.08 after SIF; cyanidin was only detected before digestion. Only hydroxybenzoic acids presented an increase throughout digestion, for instance, hydroxybenzoic acid from 3.49 ± 0.05 to 5.31 ± 0.11, and protocatechuic acid from 2.34 ± 0.11 to 7.79 ± 0.15.

The bioaccessibility (Table 4) of the bioactive compounds from EB can be perceived as the amount of the compound released inside the intestinal tract and available for assimilation. Before and after digestion, four compounds were detected, with a final bioaccessibility of 74.54 ± 5.7%.

### 2.5. The Prebiotic Potential of the Phenolic Compounds of Sambucus nigra L. Fruits

**Figure 1:**
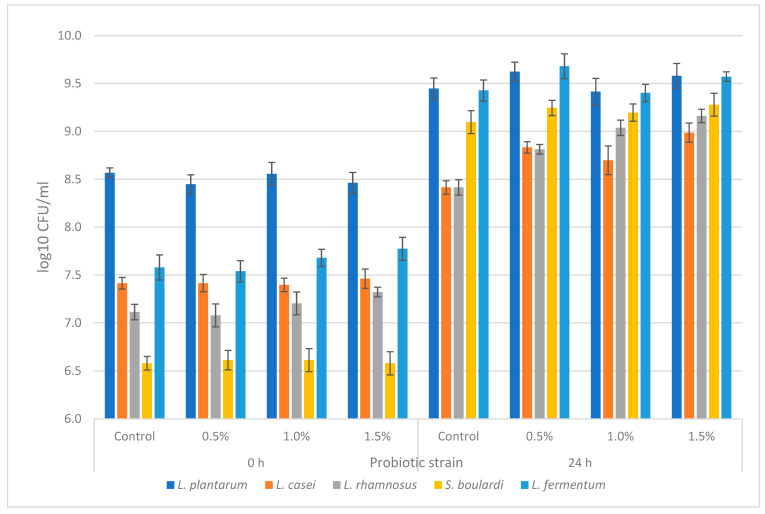
Cell viability of probiotic strains after inoculation and after 24 h (*n = 3*).

The prebiotic potential of the freeze-dried EB powder (FDEBP) was tested on the following probiotic strains: *L. plantarum*, *L. casei*, *L. rhamnosus*, *L. fermentum* and *S. boulardii*, at three different concentrations: 0.5%, 1%, and 1.5% (*w*/*v*). The cell growth of the probiotic strains was tested after inoculation and after 24 h incubation period. The results are shown in Figure 1. For the control media, we used glucose as a carbon source and the difference of log_10_ CFU/mL between incubation and inoculation was expressed as 100% GI. The influence on cell growth was most prominent in the case of *L. casei*, which yielded significant positive growth for all tested concentrations of FDEBP, with the highest results of 152.44 % GI (*p* < 0.05) recorded at 1.5% FDEBP. Moreover, positive growth was also recorded for *L. rhamnosus* and *L. plantarum*, with the highest results recorded at 141.36% (*p* < 0.05) GI with 1.5% and GI of 133.31% (*p* < 0.05) at 0.5%, respectively. In the case of *L. fermentum*, the only tested concentration that yielded a positive GI of 115.04% (*p* < 0.05) was at 0.5% FDEBP concentration. However, the least growth influence was recorded by the *S. boulardi* strain for which the 1.5% FDEBP yielded a significant GI of 107.22%. Overall, the results showed that most of the probiotic tested strains presented a positive growth influence with FDEBP as a carbon source in comparison with glucose; thus, the tested samples exhibited a prebiotic potential.

## 3. Discussion

The most significant bioactive substances found in EB in relatively high concentrations are polyphenols, which are recognized for their free radical scavenging (antioxidant) action [26]—the proprieties of each active compound being strongly correlated and induced by its unique structure [27]. As a result, most of the studies carried out on elderberries have evaluated their phenolic compound content, and thus are recognized for their multiple beneficial effects on health. There are fewer studies that follow up what happens to these bioactive compounds during digestion, and the present study proposed the quantitative and qualitative evaluation of phenolic compounds through HPLC after simulating digestion. Bioaccessibility can be defined as the amount of an ingested nutrient that is available for absorption in the gut after digestion [28] and it is an essential aspect to follow—it is known that phenolic compounds are unstable under certain conditions, and GID involves pH and temperature variations, contact with digestive enzymes, etc. [25,29].

The main polyphenols found in EB, according to published data, are chlorogenic acid, cryptochlorogenic acid, neochlorogenic acid, kaempferol-3-glucoside (astragaline), kaempferol-3-rutinoside, isorhamnetin-3-rutinoside, quercetin-3-glucoside (isoquercitrin), quercetin-3-rutinoside (rutin), quercetin. Rutin is the main flavonoid found in this plant, while EB also contains minor levels of astragaline and isoquercitrin [26]. Additionally, in our study, we identified the main phenolic compounds to be cyanidin-glucoside and cyanidin-sambubioside followed by rutin and the other compounds identified cyanidin-diglucoside, cyanidin-sambubioside-glucoside, hydroxybenzoic acid, protocatechuic acid, chlorogenic acid, caffeic acid, cyaniding, kaempferol-diglucoside, feruloyquinic acid, quercetin-glucoside and quercetin.

EBs present strong anti-inflammatory characteristics that are linked to their significant antioxidant properties. Compared to similar studies, such as by Imenšek et al. [30], who also analyzed the antioxidant activity from specific hybrids of *S. nigra*, the DPPH (96 ± 14 μmol TE/g DW) and FRAP (208 ± 25 μmol TE/g DW) results were similar; however, the ABTS values (130 ± 14 μmol TE/g DW) were higher than in our study. This study also showed the effect of maturation on the antioxidant activity of the selected plants, which indicated a growing pattern.

Data from the specialized literature attribute the antimicrobial effect to tannins and triterpenes, as well as to peptides and oligosaccharides that are present in black EB [31]. The authors of a recent study, however, draw attention to the fact that the antimicrobial activity is due to the combination of bioactive compounds from the black EB extract, rather than to certain compounds considered individually [9]. The antimicrobial activity of EB extracts has been demonstrated in several previous studies on several Gram-positive and Gram-negative bacterial strains from the following genera: *Staphylococcus*, *Pseudomonas*, *Enterococcus*, *Escherichia*, *Streptococcus*, *Klebsiella*, *Bacillus*, *Corynebacterium*, *Proteus* [9,32,33,34]. Mohammadsadeghi et al. showed that EB extract had an inhibitory effect on the development of some *Candida* species, including *Candida albicans* [32]. In none of the existing examples in the scientific literature, however, were identified studies on the antimicrobial effect on *Candida parapsilosis*, a pathogenic agent causing fungal diseases associated with significantly increased morbidity and mortality [35,36]. The present antimicrobial activity analysis results reveal a significant antimicrobial potential of EB at a concentration between 1.95 mg/mL and 3.91 mg/mL lyophilized EB powder extract. Furthermore, to our knowledge, it is the first study to demonstrate the antimicrobial effect on *C. parapsilosis*.

The bioaccessibility testing of phenolic compounds was carried out using the updated in vitro static digestion method, developed by the INFOGEST working group [37]. The in vitro digestion methods have proven to be an efficient and useful solution in anticipating the effects of in vivo digestion [38]. Anthocyanins appear to be the most unstable polyphenolic compounds during GID. In the gastric phase, the amount decreases, compared to the amount found in the extract, by 13.4%, and after intestinal digestion, the bioaccessibility of anthocyanins is 53.2%. The biggest decrease was recorded for cyanidin-glucosides and cyanidin-sambubiosides, respectively, while cyanidin was not detected by HPLC. Moreover, the data from the scientific literature mention the instability of anthocyanins in an alkaline environment [39], an aspect that is also confirmed in our study. In the intestinal tract, their hydrolysis or degradation takes place, forming phenolic acids and aldehydes. Variations at the B ring level in the structure of anthocyanins determine the degradation of cyanidin and the formation of protocatechuic acid [40]. This is one of the explanations for the spectacular changes in the amount of hydroxybenzoic acid derivatives, the subclass that became the most predominant after digestion—the total amount increased by 225% in the intestinal phase compared to the amount in the extract. The bioaccessibility of protocatechuic acid was 332.34%. Hydroxybenzoic acid showed an increase of 152.23%, the hypothesis being that HA acid can be generated either as a degradation product of anthocyanins, or as a metabolite [41,42]. Derivatives of benzoic acid have demonstrated their cardiovascular protective effects, action against cancer and obesity along with the inhibition of the inflammatory response in inflammatory bowel diseases [43,44,45].

Regarding flavonols, their presence decreases in the gastric phase compared to time 0 by 23.6%, and at the end of the intestinal phase, 60.6% of the initial amount remains. Quercetin is the flavonol that was not detected by HPLC in any of the two gastrointestinal phases. The elderberry extract contained hydroxycinnamic acids in a proportion of 18.6% of the total phenolic compounds; at the end of the gastric phase, they represented 6.95%, and 6.90% in the intestinal phase. Numerous studies carried out on polyphenols have shown a higher stability of phenolic compounds in the gastric phase with their degradation in the intestinal tract [46,47,48]. In our study, the stability of the total phenolic compounds was high (93%), and the bioaccessibility in the intestinal phase was 75%.

As there is a degree of complementarity between bioaccessibility, the prebiotic effect and overall health benefits of the bioactive compounds [49], we also monitored in the study the prebiotic potential of the phenolic compounds from black elderberry.

Prebiotics are defined as “a substrate that is selectively utilized by host microorganisms conferring a health benefit” [50], and according to the newest definition and recent studies, polyphenols were shown as a prebiotic substrate [51]. It seems that the relationship between polyphenols-intestinal microbiota is mutual: phenolic compounds can modulate the intestinal microbiota and, at the same time, microorganisms have the ability to modulate the activity of polyphenols [52]. Various preclinical studies have shown that dietary polyphenols have a prebiotic effect, stimulating the growth of different beneficial microorganisms [53,54,55]. This effect was attributed especially to anthocyanins, proanthocyanidins and catechins [56]. A previous longitudinal intervention study followed the prebiotic properties of a purified extract from EB, observing a major change in microbial diversity immediately after initiating the administration of the extract. Furthermore, in some study participants, the relative abundance of *Akkermansia* spp. increased even after supplementation was completed [22].

As far as we know, our study is among the first studies that investigated the prebiotic potential of phenolic compounds from black EB, specifically on *L. plantarum*, *L. casei*, *L. rhamnosus*, *L. fermentum and S. boulardii.* The prebiotic potential was proven on all five strains, withal, more in vitro and in vivo studies are necessary to support the findings, and to implicitly demonstrate the prebiotic potential of these compounds and their possible health-effects. However, through the results obtained regarding the prebiotic potential of the tested bacterial strains and the antimicrobial effect on *C. parapsilosis*, the current study offers new research perspectives unexplored until now, which can increase the use of bioactive potential of black EB fruits.

## 4. Materials and Methods

### 4.1. Plant Material

The present study used black *Sambucus nigra* L. fructus (elderberry) from the spontaneous flora of Romania, Bihor County (46°43′36.5″ N, 21°54′32.4″ E). The species were identified in the Pharmaceutical Botany department of the Faculty of Medicine and Pharmacy, Oradea University, Romania.

The EBs were harvested in September 2022, frozen at −20 °C and then lyophilized, using a Telstar Lyo Quest 55 plus lyophilizer (Azbil Group, Terrassa, Spain) at a temperature of −55 °C and pressure of 0.001 mbar for 72 h. Then, the lyophilized fruits were transformed by grinding and sieving into a fine powder and kept in the dark until the determinations were made.

### 4.2. Methanolic Extraction

The fine powder obtained from lyophilized EB (0.5 g) was extracted with 10 mL of methanol acidified with 1% hydrochloric acid of concentration 37% by vortexing (Heidolph Reax top, Heidolph Instruments, Schwabach, Germany) for 1 min, then sonication in an ultrasonic bath (Elmasonic E 15 H, Elma Schmindbauer, Singen, Germany) for 15 min and centrifugation (10,000 rpm for 10 min at 24 °C) in an Eppendorf AG 5804 centrifuge (Eppendorf, Hamburg, Germany). The extraction process was repeated until the complete decoloration of the sample was achieved. At the end of each extraction, the supernatant was filtered through a 0.45 µm Chromafil Xtra nylon filter (Macherey-Nagel, Duren, Germany) and combined in a flask. The obtained extract was brought to dryness by evaporating the solvent with a rotary evaporator (Rotavapor R-124, Buchi, Flawil, Switzerland) and brought back into a known volume of methanol (mL solvent retook). The extract solution was used for identifying and quantifying phenolic compounds from the lyophilized EB extract, using high-performance liquid chromatography (HPLC), respectively, for the determination of antioxidant and antimicrobial activity.

### 4.3. Qualitative and Quantitative Determinations of Phenolic Compounds Phenolic Compounds from Freeze-Dried Elderberry Extract

To identify and quantify the phenolic compounds from the lyophilized EB powder extract, an HPLC-DAD-ESI-MS system was used, consisting of an Agilent 1200 HPLC with a UV–vis detector (DAD) coupled to a mass detector (MS) with a single quadrupole Agilent 6110 (Agilent Technologies, Santa Clara, CA, USA). For the separation of phenolic compounds, the Kinetex XB C18 column (Phenomenex, Torrance, CA, USA) was used, having, as mobile phases, water + 0.1% acetic acid (solvent A), and acetonitrile + 0.1% acetic acid (solvent B), at a temperature of 25 °C, for 30 min, with a flow rate of 0.5 mL/min. The elution program was as follows: 5% B (0 min); 5% B (0–2 min); 5–40% B (2–18 min); 40–90% B; 90% B (20–24 min); 90–5% B (24–25 min); 5% B (25–30 min). For MS fragmentation, the ESI (+) ionization module was used, with a scan range between 120 and 1200 *m*/*z*, capillary voltage of 3000 V, at a temperature of 350 °C, and nitrogen flow of 7 L/min. The spectral values were recorded for all peaks in the 200–600 nm range. Phenolic compounds were identified at 280 nm, 340 nm and 520 nm. The results were analyzed using the Agilent ChemStation software (Rev B.02.01 SR2, Palo Alto, CA, USA). The phenolic compounds in the EB extract were identified considering the retention intervals; UV–vis absorption spectra and mass spectra were recorded for each peak. For the quantification of phenolic compounds, calibration curves were made with standard substances. Thus, for the quantification of the identified anthocyanins, a calibration curve was made with Cyanidin (R^2^ = 0.9951). For the quantification of hydroxybenzoic acids, the calibration curve was made with gallic acid (R^2^ = 0.9978), hydroxycinnamic acids were quantified as a chlorogenic acid equivalent (R^2^ = 0.9937), and flavonols as rutin equivalent (R^2^ = 0.9981).

### 4.4. Antioxidant Activity Assay

The antioxidant activity of the EB extract was tested using four complementary methods: DPPH, FRAP, ABTS and CUPRAC.

The DPPH (2,2-diphenyl-1-picrylhydrazyl) test was based on the ability of the compound to donate an electron (H^+^) from the structure to the DPPH radical. For the determination, the protocol previously reported by Brand-Williams et al. [57] was applied, which is the most frequently used in studies,. To summarize, lyophilized EB extract (35 μL) was mixed with 250 μL of the DPPH solution (0.02 mg/mL) and incubated for 30 min in the dark; then, the absorbance was measured at 517 nm. The resulting data were expressed as a micromole Trolox equivalent (μmol TE)/g sample.

The antioxidant method of neutralizing the ABTS radical is based on the reaction between ABTS [2,20-azino-bis(3-ethylbenzothiazoline-6-sulfonic acid)] and a compound with antioxidant activity. This reaction causes a decrease in absorbance [58]. The determination was made according to the protocol described by Arnao et al. [59], adjusted to be suitable for the 96-well microplates. In short, 20 μL of the lyophilized EB extract was mixed with 170 μL of ABTS and incubated for 6 min in the dark, then the absorbance was measured at 734 nm using a BioTek microplate reader (Synerg y HT, BioTek Instruments, Winooski, VT, USA). The resulting data were expressed as a micromole μmol TE/g sample.

The FRAP (Ferric Reducing Antioxidant Power) method is a colorimetric method that quantifies the ability of compounds with antioxidant activity to reduce (Fe^3+^) to (Fe^2+^) [60]. The assay was performed according to the protocol described by Benzie and Strain [61]. A total of 20 μL of the sample extract was added to 180 μL of the FRAP reagent. After an incubation time of 3 min, the absorbance was measured at 593 nm. The antioxidant potential was expressed as the μM Fe^2^ equivalent/g sample.

The CUPRAC test (cupric ion reducing antioxidant capacity) is a spectrophotometric technique which measures the antioxidant capacity of a compound, based on the ability of antioxidants to reduce (Cu^2+^) to (Cu^+^) [62]. The determination was performed based on the protocol described by Apak et al. [63]. The results were expressed as the μmol TE/g sample.

### 4.5. Antimicrobial Capacities

The following seven standard strains were tested: *Escherichia coli* ATCC 25922, *E. coli* ATCC 8739, *Staphylococcus aureus* ATCC 29213, *Pseudomonas aeruginosa* ATCC 27853, *Salmonella enterica* NCTC 6017, *Candida albicans* ATCC 10231 and *C. parapsilosis* ATCC 22019. They were all acquired from American Type Culture Collection (ATCC), VA, USA. The microorganisms were grown on a specific medium, Tryptic Soy agar (M1968, HiMedia Laboratories, Pvt. Ltd., Thane, India) for *E. coli* ATCC 8739, *P. aeruginosa* and both *Candida* strains, and on Mueller–Hinton agar (Oxoid Ltd., Basingstoke, Hampshire, England) for the others, within the Food Biotechnology Laboratory of the University of Agricultural Sciences and Veterinary Medicine Cluj-Napoca, Romania. The plates were incubated for 24 h at 37 °C for bacteria and 30 °C for yeasts, respectively. Bacterial and yeast morphology were confirmed by optical microscopy [64].

For each tested strain, several colonies cultivated on agar plates (Oxoid Ltd., Basingstoke, Hampshire, UK) were transferred in a sterile saline solution (8.5 g/L NaCl) and adjusted to match the turbidity of McFarland 0.5 standard which corresponded to 1.5–3 × 10^8^ CFU/mL. Then, bacterial suspensions were serially diluted 10-fold in a ratio of 1:9 in sterile serum, and 10^5^ CFU/mL solutions were added to each microplate well.

The minimum inhibitory concentration (MIC) was determined using the resazurin microtiter plate-based antibacterial assay [65,66,67]. A total of 100 µL of sterile specific growth broth medium was added to the wells of a 96-well microplate. Then, 100 µL of lyophilized EB methanolic extract (FDEBME) was added in the first well, and serial 11-fold dilutions were made in the subsequent wells of each row by transferring 100 µL from well to well. The surplus of 100 µL in the last well of the row was discarded. Then, 10 µL of appropriate inoculum was added to all wells. The positive control was gentamicin (0.4 mg/mL in saline solution). The extracts’ solvent solution (methanol: H_2_O 1:1) was added as a negative control. The microplates were incubated for 20–22 h at 37 °C or 30 °C, respectively, and then 20 µL of the 0.2 mg/mL resazurin aqueous solution was added in all wells. The microplates were subjected to a subsequent two-hour incubation. After this period, resazurin (a blue non-fluorescent dye) was oxidized to resorufin (fluorescent pink) wherever the wells contained viable bacterial cells. Thus, the concentration in the last well on each row that remained blue was considered to completely inhibit bacterial growth, the MIC. The assay was run in triplicate. The results are expressed as the mean ± standard deviation.

### 4.6. Static In Vitro Digestion of the S. nigra Samples

The updated static in vitro digestion method, developed by the INFOGEST working group, was used to simulate the GID of the samples. The protocol extensively described by Brodkorb et al. [37] is based on sequential oral, gastric and intestinal digestion. In contrast, parameters such as electrolytes, enzymes, bile, pH, dilution, and digestion time are established on available physiological data. The samples (microcapsules and lyophilized powder of *S. nigra* fruits) were subjected to a three-stage in vitro digestion process, mimicking the conditions of the mouth, stomach and small intestine. Due to the absence of starch in the matrix, the oral phase was conducted without amylase.

The samples (2 g) were diluted with 3 mL of water to achieve the proper consistency and were further diluted 1:1 (*wt*/*wt*) with simulated oral fluid (SOF) to achieve a swallowable bolus with a paste-like consistency. The SOF was composed of electrolyte solutions KCl, KH_2_PO_4_, NaHCO_3_, NaCl, MgCl_2_·6H_2_O, (NH_4_)_2_CO_3_, alongside CaCl_2_(H_2_O)_2_ and water. Further, the oral bolus was mixed with 10 mL of the simulated gastric fluid (SGF). The SGF was composed of electrolyte solutions KCl, KH_2_PO_4_, NaHCO_3_, MgCl_2_·6H_2_O, (NH_4_)_2_CO_3_, alongside CaCl_2_(H_2_O)_2_ solution (0.3 M), porcine pepsin (2000 U/mL in the final digestion mixture), and water. The pH of the samples was adjusted to 3 by adding HCl (1 M), and the mixture was homogenized and incubated for 2 h in a shaking incubator (New Brunswick Innova 44, Eppendorf AG, Hamburg, Germany). For the intestinal phase, the samples were mixed with 20 mL of pre-warmed simulated intestinal fluid (SIF) to achieve a final ratio of 1:1 (*v*/*v*). The SIF was composed of electrolyte solutions KCl, KH_2_PO_4_, NaHCO_3_, NaCl, MgCl_2_·6H_2_O, alongside CaCl_2_(H_2_O)_2_, the bile extract solution (10 mM in total digesta) and pancreatic enzymes (100 U/mL). The pH was set to 7 using NaOH (1 M), and the mixture was homogenized and incubated at 37 °C for 2 h in a shaking incubator (95 rpm). After the process was complete, 1 mL of the samples was filtered and further analyzed by HPLC to establish the bioaccessibility index.

### 4.7. The Prebiotic Potential of Phenolic Compounds of Sambucus nigra L. Fruits

To determine the prebiotic potential of the FDEBP, we tested its influence on the cell growth for the following probiotic strains: *L. plantarum* ATCC 14917, *L. casei* ATCC 393, *L. rhamnosus* LMG 25626, *L. fermentum* CECT5716 Lc40 and *S. boulardii* MYA 796, which were all acquired from the American Type Culture Collection (ATCC, Manassas, VA, USA).

The probiotic strains were obtained in freeze-dried powder form. They were activated in test tubes containing 10 mL of Man–Rogosa–Sharpe (MRS) broth (1.10661, Merck, Rahway, NJ, USA) for *Lactobacillus* strains and Potato Dextrose (PD) broth (GM403, HIMEDIA) for probiotic yeast *S. boulardii*. The tubes were incubated aerobically for 18–24 h at 37 °C in case of bacteria and at 30 °C in case of the yeast. The grown microorganisms were propagated further (10% *v*/*v*) in 100 mL flasks with 45 mL of fresh sterile media, that were used as inoculum after incubation in same conditions [68].

For the prebiotic potential assay, freeze-dried EB powder was used as the carbon source in the formulation of MRS broth and Potato Dextrose broth in three different concentrations: 0.5%, 1% and 1.5% (*w*/*v*). The control media consisted of MRS or Potato Dextrose broth with glucose as a carbon source. For the experiment, 50 mL of media in 100 mL flasks, containing either 0.5%, 1% or 1.5% of FDEBP or glucose as a carbon source, was autoclaved at 121 °C for 15 min. After that, the media was cooled to room temperature and inoculated in sterile conditions with 10% (*v*/*v*) inoculum. The inoculum used in the assay was obtained by overnight culturing in MRS broth or PD broth, respectively, for each strain. The flasks were incubated in the Heidolph 1000 shaking incubator (Heidolph Instruments, Schwabach, Germany) with 150 rpm for 24 h at 37 °C or 30 °C, respectively, in aerobic conditions.

The cell growth of the probiotic strains was tested after inoculation and after a 24 h incubation period by using the pour plate method for *Lactobacillus* strains and the spread plate method for the yeast of the serially diluted samples. Roughly 1 mL of the sample was serially diluted in 9 mL of the sterile serum solutions (0.85% *w*/*v* NaCl solution), and 1 mL of the tested dilution was pipetted in a sterile Petri dish over which 15–20 mL of semi-molten agar was poured, followed by mixing and cooling in the case of the pour plate method. For the spread plate method, 100 µL of the tested dilution was pipetted on a solidified agar plate and spread with a Driglaski spatula until complete absorption. The plates were then incubated at appropriate temperatures for 24–48 h in aerobic conditions, after which the grown colonies were counted. The experiments were run in triplicate, and the results were expressed as the mean colony-forming units CFU/mL ± standard deviation (*n = 3*). The prebiotic potential was calculated as follows: growth index GI (%) = (sample log_10_ CFU/mL 24 h − 0 h)/(control log_10_ CFU/mL 24 h − 0 h) × 100 for each strain. The difference between 24 h incubation and inoculation viability of each control was expressed as 100% GI.

## 5. Conclusions

The results show that the black elder, from the spontaneous flora of Romania, presents a high antioxidant and antimicrobial potential. Polyphenols showed a significant bioaccessibility index. During gastrointestinal digestion, anthocyanins were the most unstable polyphenolic compounds, while hydroxybenzoic acid derivatives increased significantly in the intestinal phase, compared to the amount in the extract. Moreover, this study provided, for the first time, results regarding the prebiotic potential of elderberries on *L. plantarum*, *L. casei*, *L. rhamnosus*, *L. fermentum* and *S. boulardii*, opening new directions for research and exploration of these berries. For future perspectives, in vivo studies should also be carried out in order to confirm the health benefits of elderberry.

## Figures and Tables

**Table 1:** Antioxidant capacity of *S. nigra* L. measured by different complementary assays.

Assay Method	Antioxidant Activity
DPPH (μmol TE/g DW)	104.35 ± 0.22
ABTS (μmol TE/g DW)	30.36 ± 0.18
FRAP (μmol Fe^2+^/g DW)	185 ± 0.18
CUPRAC (μmol TE/g DW)	52.3 ± 0.11

Values are expressed as mean values ± SD, *n* = 3; TE—Trolox equivalents; DW—Dry weight.

**Table 2:** Minimum inhibitory concentration against *Staphylococcus aureus*, *Salmonella enterica*, *Escherichia coli* (25922 and 8739), *Pseudomonas aeruginosa*, *Candida albicans,* and *C. parapsilosis*.

Tested Strain	*S. aureus* 25923	*S. enterica* 6017	*E. coli* 25922	*E. coli* 8739	*P. aeruginosa* 27853	*C. albicans* 10231	*C. parapsilosis* 22019
FDEBME * (mg/mL)	1.95 ± 0.1	3.91 ± 0.2	3.91 ± 0.2	3.91 ± 0.2	1.95 ± 0.1	1.95 ± 0.1	1.95 ± 0.1
Gentamicin (µg/mL)	≤0.098	≤0.098	≤0.098	12.5 ± 0.5	12.5 ± 0.5	12.5 ± 0.5	12.5 ± 0.5

* FDEBME—freeze-dried elderberry methanolic extract.

**Table 3:** The in vitro effect of gastrointestinal digestion on the phenolic content of EB mg/g.

Peak	Rt (min)	UV λmax (nm)	[M + H]^+^ (*m/z*)	Compound	Subclass	BD	SGF	SIF
1	3.81	270	139	Hydroxybenzoic acid	Hydroxybenzoic acid	3.49 ± 0.05	5.32 ± 0.14	5.31 ± 0.11
2	9.63	528, 280	611	Cyanidin-diglucoside	Anthocyanin	1.20 ± 0.07	1.14 ± 0.09	1.03 ± 0.09
			743	Cyanidin-sambubioside-glucoside				
3	10.12	295	155	Protocatechuic acid	Hydroxybenzoic acid	2.34 ± 0.11	7.32 ± 0.13	7.79 ± 0.15
4	11.09	529, 280	449	Cyanidin-glucoside	Anthocyanin	15.56 ± 0.19	13.80 ± 0.23	8.14 ± 0.08
			581	Cyanidin-sambubioside				
5	12.91	323	355	5-Caffeoylquinic acid	Hydroxycinnamic acid	1.50 ± 0.10	1.48 ± 0.14	1.28 ± 0.07
				(Chlorogenic acid)				
6	13.6	322	181	Caffeic acid	Hydroxycinnamic acid	1.27 ± 0.09	1.18 ± 0.10	0.84 ± 0.09
7	14.06	530, 280	287	Cyanidin	Anthocyanin	0.49 ± 0.03	N.D.	N.D.
8	14.47	356, 256	611	Kaempferol-diglucoside	Flavonol	0.67 ± 0.05	0.57 ± 0.04	0.45 ± 0.01
9	15.59	332	369	Feruloyquinic acid	Hydroxycinnamic acid	4.92 ± 0.11	N.D.	N.D.
10	15.88	360, 255	611	Quercetin-rutinoside	Flavonol	7.9 ± 0.09	6.30 ± 012	5.42 ± 0.16
				(Rutin)				
11	16.57	360, 255	465	Quercetin-glucoside	Flavonol	1.26 ± 0.08	1.15 ± 0.09	0.51 ± 0.08
12	21.91	360, 255	303	Quercetin	Flavonol	0.63 ± 0.03	N.D.	N.D.
				Total phenolics		41.27 ± 0.15	38.26 ± 0.21	30.76 ± 0.17

Values are expressed as mean values ± SD, *n* = 3; BD—before digestion, SGF—simulated gastric fluid, SIF—simulated intestinal fluid; N.D.—not determined.

**Table 4:** The bioaccessibility of EB extract.

Compound	Bioaccesibility (%)
Anthocyanins	53.17 ± 1.5
Flavonols	60.64 ± 3.8
Hydroxycinnamic acids	27.59 ± 2.1
Hydroxybenzoic acid	224.64 ± 5.8
Total phenolics	74.54 ± 5.7

Values are expressed as mean values ± SD.

## Data Availability

Data are contained within the article.

## Sample Availability

Samples of the compounds are available from the authors.
